# Drug‐Induced Lung Injury Masking Lung Adenocarcinoma

**DOI:** 10.1002/rcr2.70678

**Published:** 2026-07-17

**Authors:** Keisuke Watanabe, Toshiko Kamata, Shigetoshi Yoshida, Tetsuo Sato

**Affiliations:** ^1^ Department of Pulmonary Medicine International University of Health and Welfare Atami Hospital Atami Shizuoka Japan; ^2^ Department of Thoracic Surgery International University of Health and Welfare Atami Hospital Atami Shizuoka Japan; ^3^ Department of Thoracic Surgery International University of Health and Welfare School of Medicine Chiba Japan

**Keywords:** computed tomography, drug‐induced lung injury, lung cancer, pneumonic‐type lung adenocarcinoma

## Abstract

We report a case of lung adenocarcinoma masked by drug‐induced lung injury. Lung adenocarcinoma can present with consolidation, known as pneumonic‐type lung adenocarcinoma, which mimics inflammatory lung disease. Therefore, careful radiological follow‐up should be performed even after apparent resolution of pneumonia.

## Graphical abstract

After resolution of the drug‐induced lung injury, the lesion in the left upper division persisted, and a left segmentectomy was performed. The final pathological diagnosis was lung adenocarcinoma.

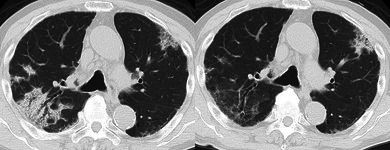

## 1 Clinical Image

An 82‐year‐old man was referred to our department with a 2‐week history of dyspnea. His medical history included haemorrhoids, for which he had been taking a Kampo (Japanese herbal) medicine for 2 or 3 months. His vital signs were stable, and fine crackles were heard over the dorsal lung fields. Chest computed tomography (CT) revealed bilateral infiltrative shadows (Figure 1A,B), and drug‐induced lung injury was suspected. The Kampo medicine was discontinued. Follow‐up CT performed 2 months after discontinuation of the Kampo medicine showed improvement of the infiltrative shadow; however, the lesion in the left upper division persisted (Figure 2A,B). Review of previous CT scans revealed gradual enlargement of the lesion, raising suspicion of lung cancer. A left segmentectomy was performed, and the final pathological diagnosis was lung adenocarcinoma (Figure 3A,B). Lung adenocarcinoma can present with consolidation, known as pneumonic‐type lung adenocarcinoma, which mimics inflammatory lung disease [1]. Therefore, careful radiological follow‐up should be performed even after apparent resolution of pneumonia.

**FIGURE 1:**
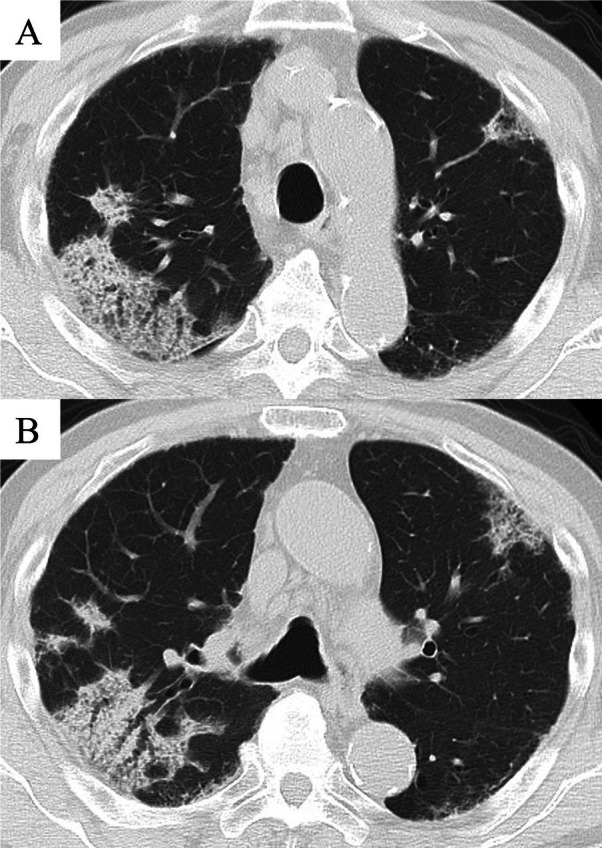
Chest computed tomography revealed bilateral infiltrative shadows (A, B).

**FIGURE 2:**
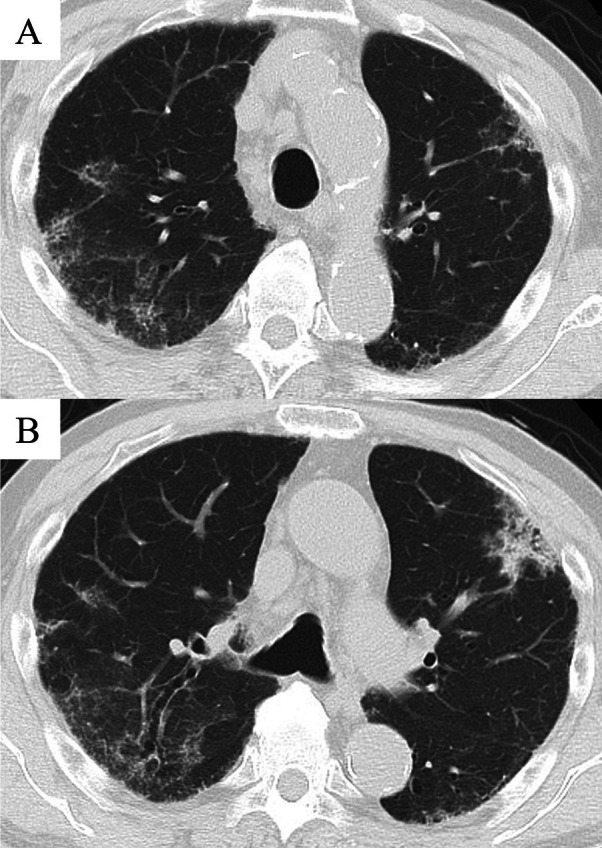
Follow‐up chest computed tomography showed improvement of the infiltrative shadow; however, the lesion in the left upper division persisted (A, B).

**FIGURE 3:**
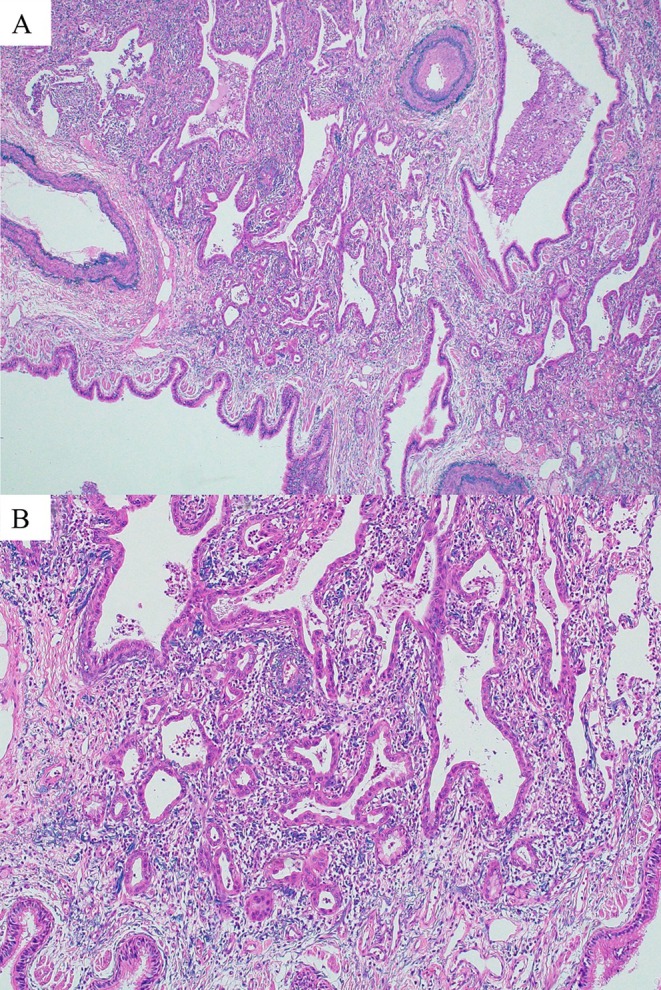
Pathological findings of lung adenocarcinoma. Haematoxylin and eosin staining (A, ×40; B, ×100).

## Author Contributions

Keisuke Watanabe contributed to the conception and design of the work, the acquisition, analysis and interpretation of data for the work; drafting the work; and final approval of the version to be published. Toshiko Kamata, Shigetoshi Yoshida and Tetsuo Sato contributed to the conception of the work, the acquisition, analysis or interpretation of data for the work; reviewing it critically for important intellectual content; and final approval of the version to be published.

## Funding

The authors have nothing to report.

## Consent

The authors declare that written informed consent was obtained for the publication of this manuscript and accompanying images and attest that the form used to obtain consent from the patient complies with the Journal requirements as outlined in the author guidelines.

## Conflicts of Interest

The authors declare no conflicts of interest.

## Data Availability

The data that support the findings of this study are available on request from the corresponding author. The data are not publicly available due to privacy or ethical restrictions.
